# Torsion of a Wandering Spleen

**DOI:** 10.4103/1319-3767.70618

**Published:** 2010-10

**Authors:** Hicham El Bouhaddouti, Jihane Lamrani, Abdellatif Louchi, Mounia El Yousfi, Noureddine Aqodad, Adil Ibrahimi, Meriem Boubou, Imane Kamaoui, Siham Tizniti

**Affiliations:** Department of Digestive Surgery, University Hospital Hassan II Fes, Morocco; 1 Department of Gastroenterology, University Hospital Hassan II Fes, Morocco; 2 Department of Medical Imaging, University Hospital Hassan II Fes, Morocco

**Keywords:** Splenopexy, splenectomy, wandering spleen

## Abstract

Wandering spleen is a rare condition defined as a mobile spleen only attached with its pedicle. It can be complicated by a volvulus, which is a surgical abdominal emergency. Preventing infarction is the aim of a prompt surgery that can preserve the spleen and then proceed to splenopexy. We report a rare case of torsion of a wandering spleen associated with a dolichosigmoïd.

Wandering spleen is a rare condition characterized by the absence or underdevelopment of one or all of the ligaments that hold the spleen in its normal position in the left upper quadrant of the abdomen.[1] It is an uncommon clinical entity. It mainly affects children, who make up one third of all cases[2] with a female predominance after the age of one. At adult age it most frequently affects women of reproductive age, in whom acquired laxity of the splenic ligaments is usually the cause.[2] The clinical presentation of wandering spleen is variable, but the main symptom is abdominal pain. Its major complication is acute torsion with subsequent infarct, which is a potentially fatal emergency. We report a case of torsion of a wandering spleen associated with a dolichomegasigmoïd.

## CASE REPORT

**Figure 1:**
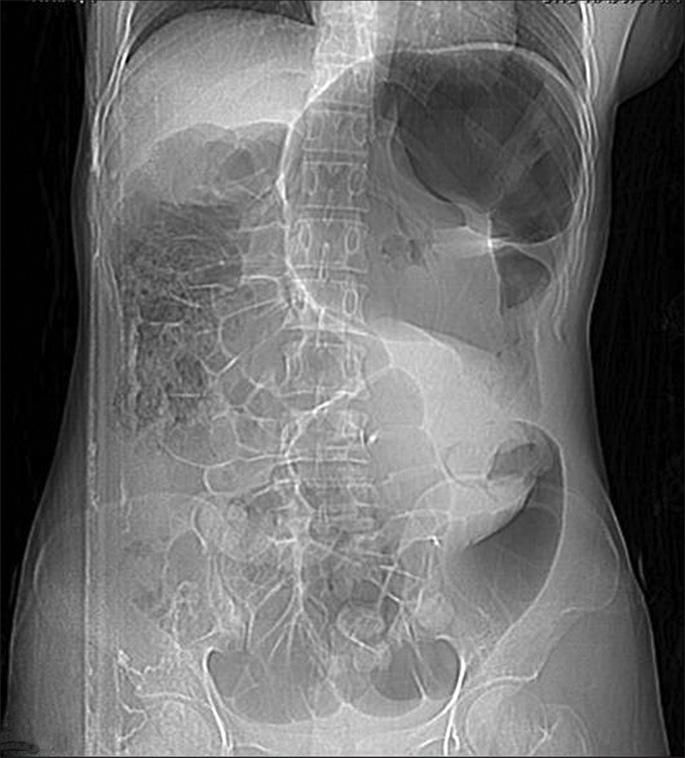
Abdominal radiography showed an important colon distention at the upper left quadrant without air fluid levels

**Figure 2:**
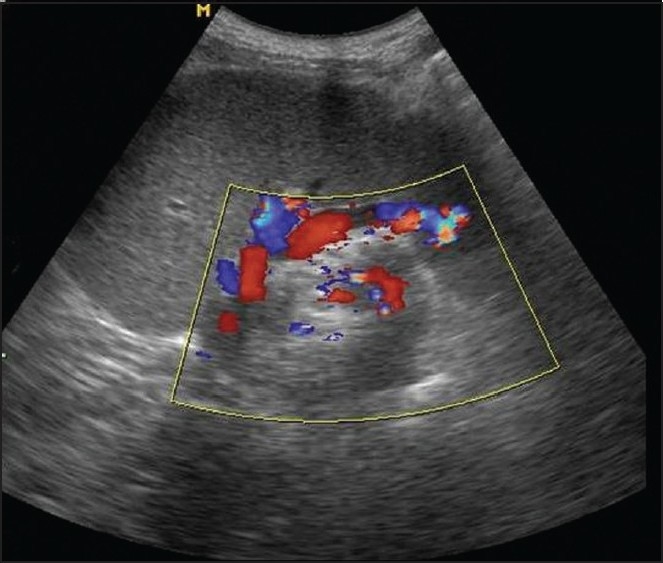
Ultrasonography image demonstrated a whorl of concentric vessels in the region of splenic hilum. Note a spleen of ectopic position

**Figure 3:**
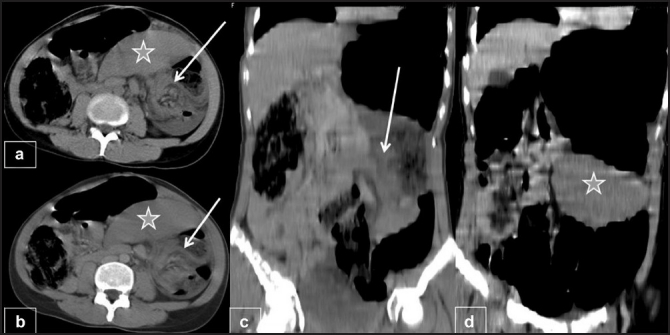
Contrast enhanced abdominal CT in axial (a, b) and sagittal views (c, d) showed spleen vessels and surrounding fat forming a whirled appearance (arrows) at the spleen (star) hilum. The spleen is located below the level of the left kidney

**Figure 4:**
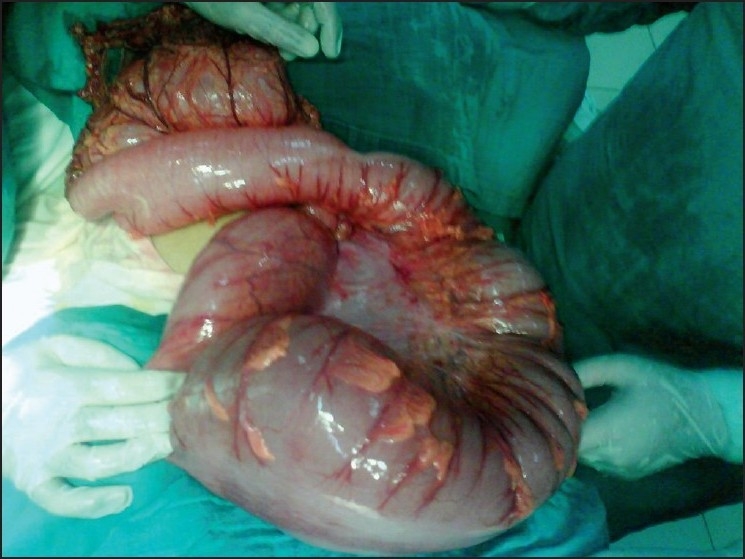
Dolichosigmoid associated to the wandering spleen

**Figure 5:**
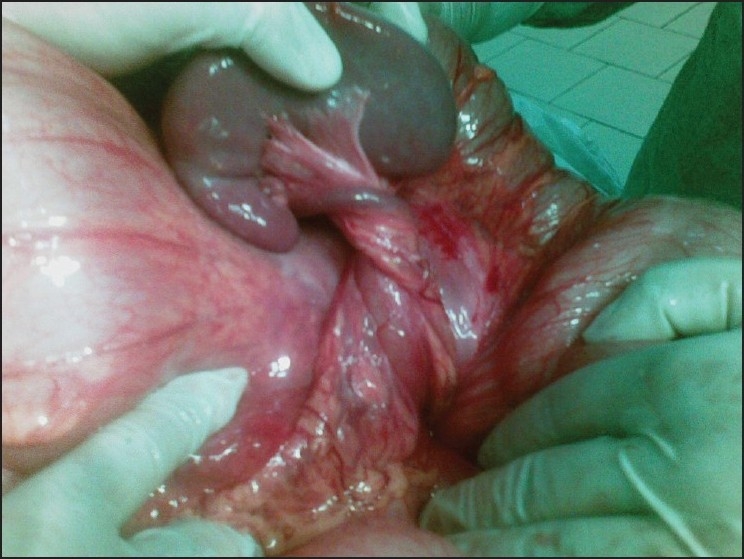
Volvulus of the wandering spleen

A 27-year-old woman presented to the emergency department after three days of abdominal pain, vomiting and constipation without fever. She had a recurrent constipation but no history of recurrent abdominal pain, abdominal mass, or trauma. On physical examination, abdominal distension was noted. There was a moderate diffuse abdominal tenderness more pronounced on the left side. Abdominal radiography showed an important colon distention especially at the upper left quadrant without air fluid levels [Figure 1]. An abdominal ultrasonography showed a “whirl” image beside a spleen of ectopic position with homogenous echo texture [Figure 2]. Computed tomography (CT) revealed a colonic distention, especially of the sigmoid and an abnormal position of the spleen at the left lower quadrant without any sign of spleen ischemia [Figure 3]. The patient underwent laparotomy. Her spleen was in an ectopic position. It was rounded by a large and long left colon [Figure 4]; there was no sustentaculum lieni, nor any of the spleen ligaments. The spleen pedicle was twisted without any sign of ischemia [Figure 5]. Splenopexy was performed by the fixation of the spleen pedicle to the posteroparietal peritomeum. We used separated sutures with vicryl^®^ 0 prepared on the upper and lower borders of the pedicle before the spleen replacement in its quadrant, which were knotted at the end; sigmoidectomy and colorectal anastomosis were performed. The patient recovery was uneventful. She was discharged on the sixth day.

## DISCUSSION

Wandering spleen is defined as mobile spleen that is attached only by an elongated vascular pedicle, allowing it to migrate to any part of the abdomen or pelvis. It is a result of congenital anomalies in the development of the dorsal mesogastrium and the absence or malformation of normal splenic suspensory ligaments.[1,3] The splenic ligaments include the gastrolienal and lienorenal ligaments. The former attaches the spleen to the greater curvature of the stomach, whereas the latter attaches the spleen to the posterior abdominal wall, both ligaments attach to the hilum of the spleen medially. The phrenicocolic ligament supports the spleen inferiorly.[3]

However, acquired anomalies have been described and are attributed to laxity of the ligaments due to weakness of the abdominal wall, multiple pregnancies, hormonal changes or increase in size in the spleen.[4] Both congenital and acquired conditions result in a long pedicle, which is predisposed to torsion. The splenic vessels course within the pedicle, and therefore, torsion of the pedicle results in partial or complete infarct of the spleen.

Patients with a wandering spleen may be asymptomatic, present with a movable mass in the abdomen, or have chronic or intermittent abdominal pain because of partial torsion and spontaneous detorsion of the spleen.[5,6] Torsion is the most common complication.[4] It usually presents as an acute abdominal problem. This makes the physical examination more difficult and preoperative diagnosis less accurate. Clinically, the diagnosis can be suspected when a firm, movable abdominal mass is felt with the typically described “notched border”. However splenic engorgement may hide the splenic notch.[7,8] Preoperative diagnosis of wandering spleen is rarely suggested, based on clinical findings alone, because of nonspecific symptoms. Therefore, imaging plays a major role in establishing the diagnosis,[6,8] plain radiography and barium studies showed medial or superior displacement with extrinsic impression of the splenic flexure of colon along with a soft tissue mass in an unusual site corresponding to the wandering spleen[5] but are non-specific.

Sonography showed the characteristic comma-shaped spleen in an ectopic position and the lack of splenic tissue in the left upper quadrant. Duplex Doppler color flow evaluation, provided optimal visualization of the organ and assessment of vascular supply.[8] However, sonography can often be hampered by bowel gas. Angiography can also provide definite evidence of splenic torsion and ectopic splenic location, showing a tapered and abruptly twisted distal splenic artery at the point of torsion, but it is invasive and not essential for diagnostic purposes.[1,6]

Computed tomography is the preferred study for diagnosing a wandering spleen when torsion is suspected clinically or on other imaging studies. The CT manifestations included: (I) absence of the spleen anterior to the left kidney and posterior to the stomach, (II) a lower abdominal or pelvic mass with homogenous or heterogenous splenic parenchyma and an attenuation value less than that of normal splenic tissue, (III) whorled appearance of splenic vessels and surrounding fat only, and (IV) secondary findings such as ascites and necrosis of the pancreatic tail.[5,7,9]

However, it is the whorled appearance of the splenic vessels and surrounding fat at the splenic hilum that is considered as specific of torsion of a wandering spleen.[10] This sign was shown by the CT scan of the patient of this case. Until recently, splenectomy has been performed for wandering spleen[11,12] though several authors had advocated splenopexy earlier. Stringel *et al*.[4] fixed the spleen via its pedicle. Maxwell-Armstrong *et al*.[13] fixed it by omentum. Caracciolo *et al*.[14] and Peitgen *et al*.[15] used transposition of the colonic flexure and gastrocolic ligament for splenopexy. Allen and Andrews[16] sutured a basket of Dexon® mesh around the spleen, whereas Seashore and McIntosh,[17] van der Staak and Festen,[18] and Steinberg *et al*.[19] dissected a posterolateral retroperitoneal pouch during laparotomy. Actually, laparoscopic splenopexy is an easy alternative to laparotomy. The laparoscopic approaches reported[18,20] have used sacks and slings of Dexon® or Vicryl® mesh for fixation or an autologous peritoneal pouch in the posterolateral abdominal wall permitting to avoid the risk of infection of the mesh. The latest technique seems to have the best results according to the satisfaction of the patients and the esthetic appearance.[21]

In the present case, the spleen was fixed by its pedicle using a technique inspired by the one described by stringel.[4] In the stringel technique, the spleen was repositioned in the splenic fossa in the left upper quadrant. Two stabilizing continuous sutures of 3/0 silk were inserted running from the upper and lower ends of the hilum of the spleen to the posteroparietal peritoneum. These sutures appeared to provide enough fixation of the spleen to prevent torsion.[4]

The splenic torsion is sometimes associated with other manifestations like gastric or pancreatic tail volvulus.[22,23] The only colonic manifestations reported is obstruction by splenic flexure volvulus.[24,25] Dolichosigmoid associated with a wandering spleen seems to be reported for the first time in our case.

## CONCLUSION

The torsion of a wandering spleen is a rare abdominal emergency. Its diagnosis should be made in prompt time to prevent infarction of the spleen. Splenopexy by means of the extraperitoneal pocket creation appears to allow anatomic placement of the spleen along with the protection of the rib cage without the employment of biomaterials. Splenectomy should be performed only in patients with splenic torsion in whom massive infarction and thrombosis of the splenic vessels has occurred.
